# A systematic review of non-motor symptom evaluation in clinical trials for amyotrophic lateral sclerosis

**DOI:** 10.1007/s00415-021-10651-1

**Published:** 2021-06-13

**Authors:** Emily Beswick, Deborah Forbes, Zack Hassan, Charis Wong, Judith Newton, Alan Carson, Sharon Abrahams, Siddharthan Chandran, Suvankar Pal

**Affiliations:** 1 Centre for Clinical Brain Sciences, The University of Edinburgh, Edinburgh, Scotland, UK; 2 Anne Rowling Regenerative Neurology Clinic, The University of Edinburgh, 49 Little France Crescent, Edinburgh, EH16 4 SB Scotland, UK; 3 Euan MacDonald Centre for MND Research, The University of Edinburgh, Edinburgh, Scotland, UK; 4 Human Cognitive Neurosciences, Psychology, School of Philosophy, Psychology and Language Sciences, The University of Edinburgh, Edinburgh, Scotland, UK; 5 UK Dementia Research Institute, The University of Edinburgh, Edinburgh, Scotland, UK

**Keywords:** Amyotrophic lateral sclerosis, Clinical trials, Non-motor symptoms, Outcome measures

## Abstract

**Background:**

Amyotrophic lateral sclerosis (ALS) is increasingly recognised as a multi-system disorder, presenting with common and impactful non-motor symptoms, such as neuropsychiatric symtpoms, cognitive and behavioural changes, pain, disordered sleep, fatigue and problematic saliva.

**Aim/hypothesis:**

We aimed to systematically review 25 years of ALS clinical trials data to identify if non-motor features were evaluated, in addition to the traditional measures of motor functioning and survival, and where evaluated to describe the instruments used to assess. We hypothesised that assessment of non-motor symptoms has been largely neglected in trial design and not evaluated with ALS-suitable instruments.

**Methods:**

We reviewed clinical trials of investigative medicinal products in ALS, since the licensing of riluzole in 1994. Trial registry databases including WHO International Trials Registry, European Clinical Trials Register, clinicaltrials.gov, and PubMed were systematically searched for Phase II, III or IV trials registered, completed or published between 01/01/1994 and 16/09/2020. No language restrictions were applied.

**Results:**

237 clinical trials, including over 29,222 participants, were investigated for their use of non-motor outcome measures. These trials evaluated neuropsychiatric symptoms (75, 32%), cognitive impairment (16, 6.8%), behavioural change (34, 14%), pain (55, 23%), sleep disturbances (12, 5%) and fatigue (18, 8%). Problematic saliva was assessed as part of composite ALS-FRS(R) scores in 184 trials (78%) but with no focus on this as an isolated symptom. 31 (13%) trials including 3585 participants did not include any assessment of non-motor symptoms.

**Conclusions:**

Non-motor symptoms such as neuropsychiatric, cognitive and behavioural changes, pain, disordered sleep, fatigue, and problematic saliva have not been consistently evaluated in trials for people with ALS. Where evaluated, non-symptoms were primarily assessed using instruments and impairment thresholds that are not adapted for people with ALS. Future trials should include non-motor symptom assessments to evaluate the additional potential therapeutic benefit of candidate drugs.

**PROPSERO registration:**

CRD42020223648.

**Supplementary Information:**

The online version contains supplementary material available at 10.1007/s00415-021-10651-1.

## Introduction

The focus of assessment and symptom management in amyotrophic lateral sclerosis (ALS) is traditionally on limb weakness, speech and swallowing difficulties, and respiratory failure. Despite this, a range of other symptoms are repeatedly reported as impactful and poorly evaluated in people with ALS (pwALS) including neuropsychiatric symptoms, cognitive and behavioural changes, pain, disordered sleep, fatigue, and problematic saliva [1].

These symptoms are often collectively termed ‘non-motor’ or ‘extra-motor’ [1, 2] and result in significant functional impairment, reduced quality of life (QoL), higher disease burden, and negative prognoses [2–6]. People with ALS experiencing greater frequency of non-motor symptoms report lower quality of life than those who indicate more severe motor symptoms, suggesting that the impact of these non-motor symptoms on the daily lives of people with ALS is comparable to, if not greater, than of motor symptoms [2, 3]. These symptoms can arise secondary to motor dysfunction, such as inefficient saliva clearance from bulbar motor dysfunction, and pain from inability to regularly move and turn. Symptoms may localise elsewhere neuroanatomically [2] broadening our understanding of the aetiopathogenesis of ALS and providing insights into wider neuroanatomical dysfunction [7]. Clinical management [8] and trial design guidelines for ALS [9] have evolved to incorporate evaluation and treatment of non-motor symptoms as part of holistic assessment of ALS [10].

Our previous work has reported how neuropsychiatric, cognitive and behavioural assessments have been employed as outcome measures and exclusion criteria in ALS trials [11]. This identified that these aspects were under-evaluated in trial design, and often using measures unsuitable, or not adapted for, people with progressive disability. In this study, we intend to broaden our scope to include other non-motor symptoms important in ALS: pain, sleep disturbance, fatigue, and problematic saliva. In addition, we will evaluate the assessment tools used as outcome measures in greater detail, we have continued to include neuropsychiatric, cognitive and behaviour assessment in this review to provide a complete picture of non-motor evaluation.

### Non-motor symptoms in clinical care and trial design

The focus on motor symptoms in clinical and research contexts is likely impacted by the limited availability of disease-modifying drugs for people with ALS. Riluzole is currently the only globally licensed disease-modifying therapy for ALS with limited efficacy, resulting in prolongation of survival by 2–3 months [12]. There is a significant unmet need in therapeutic options for people affected by ALS.

To deliver holistic disease management for people with ALS, it is necessary to expand our conceptualisation of ‘treatment’ beyond improved physical function and extended survival. Effective management, or ultimately slowed progression, of non-motor symptoms due to pharmacological intervention should be evaluated as part of any novel investigative medicinal products’ efficacy in clinical trials [13]. Inclusion of alternative outcome measures to evaluate potential impact of candidate drugs on non-motor impairment is recommended as a potential area of consideration for trial design in the current Airlie House guidelines, which focus on ALS-specific trial development [9]. The potential beneficial effect of candidate drugs which successfully manage non-motor features of a debilitating condition may have significant clinical impact, improving quality of life, reducing disability and disease burden.

The method of assessment is also of particular relevance in trials of people with ALS. Due to progressive disability, overlap with somatic symptoms, disease-specific impairments and speech decline, traditional measures may not be as effective in detecting change in symptoms, directly reducing their suitability to evaluate people with ALS [1]. This can be mediated through using tools which are validated specifically for this cohort [14, 15], or tools with revised impairment thresholds [16] which account for the specific profile of impairment characterised by ALS. In this systematic review of non-motor outcome measures in ALS trials, we will consider the types of assessment tools used and their suitability to evaluate non-motor presentations in this population.

### Aims and hypotheses

We aimed to systematically review historical clinical trials of interventional medicinal products (IMPs) in ALS, since the licensing of riluzole in 1994, to identify if non-motor features of ALS were evaluated as outcome measures. In addition, we aimed to review the assessment tools used, their characteristics and suitability for evaluating non-motor symptoms in people with ALS. We hypothesised that non-motor symptoms have been largely overlooked in trial design and that where evaluated, assessed with instruments that are not specifically designed to evaluate symptoms in this population.

## Methods

We completed a systematic, unbiased, search of trial registries including clinicaltrials.gov, World Health Organisation’s (WHO) International Clinical Trials Registry Platform (ICTRP), European Union Clinical Trials Register (EduraCT) and PubMed on 16/09/2020 for Clinical Trials of an Investigational Medicinal Product (CTIMP). Using the search terms “amyotrophic lateral sclerosis” or “motor neuron* disease”, we searched clinicaltrials.gov for interventional trials of investigative medicinal products. We searched European Union Clinical Trials Register (EudraCT) and WHO International Clinical Trials Registry Platform (ICTRP) for trials of “amyotrophic lateral sclerosis” with the filters “Phase II”, “Phase III” and “Phase IV” applied. Using the advanced search feature, we filtered PubMed with (“amyotrophic lateral sclerosis”[MeSH Terms] OR “motor neuron* disease” [MeSH Terms]). We then applied the ‘Clinical Trial’ filter for Article Type, Human trials only and Publication Date within the criteria defined above.

Phase II, III or IV trials assessing potential disease-modifying therapies in subjects with amyotrophic lateral sclerosis that were registered, completed or published between 01/01/1994 and 16/09/2020 were included. No language restrictions were applied. Extension trials, post hoc analysis papers, stem cell therapies, imaging studies, medical device studies, non-ALS subjects and trials focussed on symptom management were excluded.

### Data extraction

The following details of selected trials were extracted “Investigational Medicinal Product (IMP) Assessed”, “Number of Participants”, “Date of Commencement”, “Primary Outcome Measure(s)” and “Secondary Outcome Measure(s)”. We reviewed each assessment tool used as an exclusion criteria or outcome measure in the included trials to explore whether they evaluated non-motor symptoms; defined in this study as neuropsychiatric, cognitive impairment, behavioural changes, pain, disordered sleep, fatigue, and problematic saliva. Each assessment tool was categorised as ALS specific, symptom specific, both ALS and symptom specific or generic in content focus. We then reviewed each trial included in this review for their use of each assessment tool and subsequent evaluation of each non-motor symptom.

Each assessment tool was reviewed and data extracted on the intended focus of assessment, administrator (clinician or self-report), if the scoring is affected by the presence of motor disability or speech impairment and the time to administer. We also explored the availability of disease-specific impairment thresholds where applicable, and the availability of non-English translations.

## Results

### Overview

**Fig. 1:**
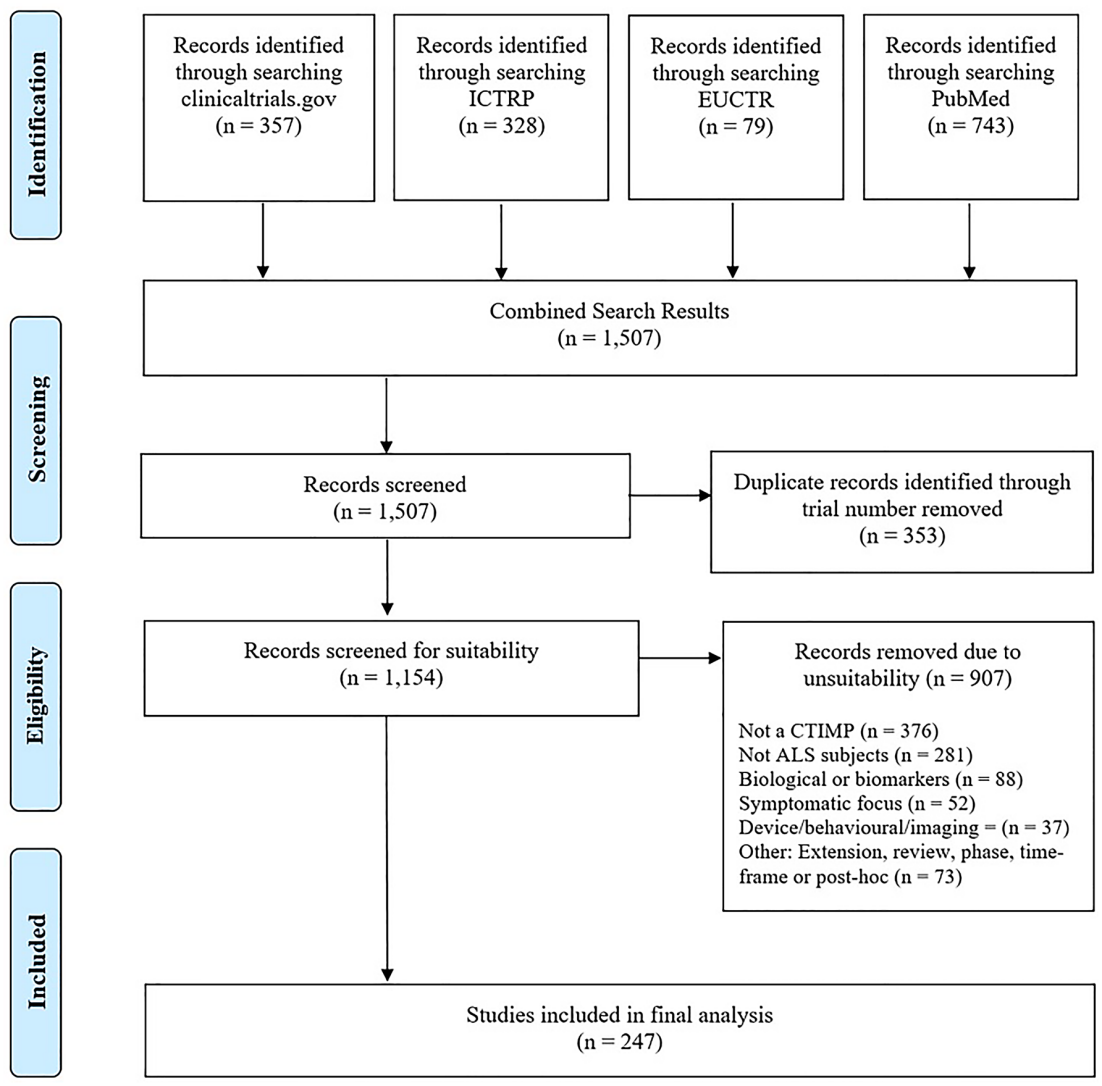
PRISMA diagram for record selection process

The search identified 1507 records, (PRISMA diagram in Fig. 1). 353 were removed due to duplication and 907 did not meet inclusion criteria (defined in Fig. 1); in particular results which were not clinical trials of investigative medicinal products and non-ALS subjects. 237 clinical trials remained. These trials were proposed to include over 29,222 trial participants with ALS. The non-motor symptoms evaluated in this review are neuropsychiatric, cognitive impairment, behavioural changes, pain, disordered sleep, fatigue, and problematic saliva. Table 1 summarises the reported prevalence of these symptoms in the ALS population, and the pharmacological and non-pharmacological treatments suggested with evidence derived from a Cochrane database systematic review of treatments [10] and United Kingdom National Institute for Clinical Excellence (NICE) clinical care guidelines [8]. The trials forming the main dataset of this review are focussed on therapeutic targets for motor symptoms and survival improvement. Table 2 provides a summary of how frequently each non-motor symptom considered in this review was evaluated in the clinical trials. These seven non-motor symptoms were included as outcome measures or evaluated within quality of life measures (QoL) in 206 trials (87%). Neuropsychiatric symptoms were assessed in 75 trials (32%) and cognitive impairment was evaluated as an outcome measure in 16 trials (6.8%) Behavioural change was evaluated in 33 trials (14%), pain in 55 trials (23%) and fatigue in 18 trials (8%). Sleep disturbances were evaluated in 12 trials (5%). Whilst saliva assessment was included in 184 trials (78%), this was part of a composite measure embedded within the either the ALS-FRS-(R) (Amyotrophic Lateral Sclerosis Functional Rating Scale Revised [17]) or the CNS-BFS (Centre for Neurologic Studies Bulbar Function Scale [18]), and the impact of drugs of saliva problems was not assessed specifically. 31 trials (13%) did not include any assessment of saliva, neuropsychiatric, cognitive impairment, behavioural changes, pain, disordered sleep and fatigue as an outcome measure or evaluate within a quality of life measure.

**Table 1:** Non-motor symptoms and potential treatments in ALS

Symptom	Details	Reported prevalence	Non-pharmacological interventions	Pharmacological interventions	References
Neuropsychiatric	Depression	Depression 0–44%	Talking therapies	Benzodiazepines and other anxiolytic medications	[46, 51–54]
	Anxiety	Anxiety 0–33%	Music therapy	Antidepressants (particularly the SSRI and TCA groups)	
	Suicidal ideation	Suicidal ideation 13.3%	Hypnosis	Dextromethorphan and quinidine combination for emotional lability	
	Emotional lability	Emotional lability 50%	Mindfulness	Amitriptyline due to additional anticholinergic effects on excessive saliva and insomnia	
			Cognitive behavioural therapy		
Cognitive impairment	Executive dysfunction Social cognition Verbal fluency	30–50% of pwALS have some level of cognitive impairment	Trial of non-invasive ventilation (NIV) to evaluate if cognitive problems may be related to respiratory impairment	No medications to address AL specific cognitive impairment currently available Cognitive status must also be considered in prescribing symptom management drugs	[8, 10, 55–57]
	Working memory Language dysfunction	6–14% of pwALS reach threshold for FTD		Glycopyrrolate as first option of treating sialorrhea in pwALS with cognitive impairment as this has fewer central nervous system side effects	
Behavioural change	Apathy Impulsivity Disinhibition Perseveration Loss of Sympathy/empathy Hyper-orality and change in eating behaviour	24–69% of pwALS experience some behavioural change Apathy is most common behavioural change, experienced by 28% of pwALS	No ALS-specific but FTD interventions available which may be beneficial Environmental management (reducing noise, clutter and stimuli and avoiding potentially problematic situations) Non-verbal cues Creating reward systems	Benzodiazepines	[55, 56, 58]
Pain	Spasticity Cramps Joint immobility Pressure sores	Up to 57% of people with ALS report pain	Exercise programmes (physiotherapy)	Cramps Quinine Levetiracetam Mexiletine Spasticity Nonsteroidal anti-inflammatories Muscle relaxants Baclofen, tizanidine or dantrolene Intrathecal baclofen	[10, 46, 59–61]
Sleep disturbances	Poor quality sleep Difficulty getting to, or staying, asleep Daytime sleepiness	Exact prevalence unknown due to multiple potential aetiologies of sleep problems but 45–60% of pwALS report disturbed sleep due to breathing problems	Non-invasive ventilation	Amitriptyline (antidepressant with additional benefit of insomnia management) Sedatives (opioids and benzodiazepines)	[10, 62–65]
Fatigue	Mental fatigue Physical fatigue	44% of pwALS experience clinically significant fatigue and this is associated with disease severity	Resistance exercise Respiratory exercise Repetitive transcranial magnetic stimulation (rTMS)	Modafinil	[8, 10, 66, 67]
Saliva	Excessive oral secretion Dry mouth	Problematic saliva 37.5%	Radiotherapy Suction Humidification of NIV	Hyoscine patches Amitriptyline Atropine drops Glycopyrrolate Carbocisteine Botulinum toxin	[8, 10, 46, 68, 69]

**Table 2:** Assessments utilised and non-motor symptoms evaluated

Tool	Intended area of focus	Non-motor symptom assessed	Separate score for non-motor symptom?	ALS specific	Symptom specific	ALS and symptom specific	Frequency of use as an outcome measure^a^
C-SSRS (Columbia Suicide Severity Rating Scale)	Suicidality	Neuropsychiatric	Yes	No	Yes	No	4
NPI-Q (Neuropsychiatric Inventory Questionnaire)	Neuropsychiatric	Neuropsychiatric	Yes	No	Yes	No	1
ESS (Edmonton Symptom Assessment Scale)	Quality of life	Neuropsychiatric	No	No	No	No	1
		Pain	No	No	No	No	1
		Sleep	No	No	No	No	1
HADS (Hospital Anxiety and Depression Scale)	Anxiety and depression	Neuropsychiatric	Yes	No	Yes	No	2
ADI-12 (Amyotrophic Lateral Sclerosis Depression Inventory-12 item)	Depression	Neuropsychiatric	Yes	No	No	Yes	1
HAM-D (Hamilton-Depression)	Depression	Neuropsychiatric	Yes	No	Yes	No	3
ALSSQOL-R (Amyotrophic Lateral Sclerosis Specific Quality of Life—Revised and Short Form)	Quality of life	Pain	No	Yes	No	No	33
		Fatigue	No	Yes	No	No	33
		Neuropsychiatric	No	Yes	No	No	33
		Sleep	No	Yes	No	No	33
		Cognition^b^	No	Yes	No	No	33
SF-8, SF-12 and SF-36 (Short Form Health Survey—8 item, 12 item or 36 item)	Quality of life	Pain	No	No	No	No	31
		Neuropsychiatric	No	No	No	No	31
SEI-QoL (Schedule for Individual Quality of Life)	Quality of life	Self-reported (any)	No	No	No	No	1
ALSAQ-5 (Amyotrophic Lateral Sclerosis Assessment Questionnaire—5 item)	Quality of life	Neuropsychiatric	No	Yes	No	No	6
ALSAQ-40 (Amyotrophic Lateral Sclerosis Assessment Questionnaire—40 item)	Quality of life	Pain	No	Yes	No	No	34
		Neuropsychiatric	No	Yes	No	No	34
EQ-5D-5L and EQ-5D-3L (Europol—5 Dimension—5 and 3 Level)	Quality of life	Pain	No	No	No	No	13
		Neuropsychiatric	No	No	No	No	13
McGill or McGill Revised	Quality of life	Neuropsychiatric	No	No	No	No	9
KFSS (Krupp Fatigue Severity Scale)	Fatigue	Fatigue	Yes	No	Yes	No	3
VAS (Visual Analog Scale)	General	Fatigue	Yes	No	No	No	5
		Pain (cramp)	No	No	No	No	7
		Sleep	No	No	No	No	1
		Behavioural (emotionality)	No	No	No	No	1
		Saliva	No	No	No	No	1
SIP (Sickness Impact Profile)/ALS-19	General	Behaviour	No	No	No	No	5
		Neuropsychiatric	No	No	No	No	5
		Sleep	No	No	No	No	5
ALS-FRS-(R) (Amyotrophic Lateral Sclerosis Functional Rating Scale)	Physical function	Saliva	No	Yes	No	No	182
ESS (Epworth Sleepiness Scale)	Sleep	Sleep	Yes	No	Yes	No	2
PSQI (Pittsburgh Sleep Quality Index)	Sleep	Sleep	Yes	No	No	Yes	1
Norris Scale	Physical functioning	Behavioural	No	Yes	No	No	18
ECAS (Edinburgh Cognitive Assessment Screen)	Cognition and behavioural change	Behaviour	Yes	No	No	Yes	14
		Cognition	Yes	No	No	Yes	14
FBI (Frontal Behavioural Inventory)	Behavioural change	Behaviour	Yes	No	Yes	No	1
ACE-III (Addenbrooke’s Cognitive Examination—III)	Cognition	Cognition	Yes	No	Yes	No	2
ALS-CBS (Amyotrophic Lateral Sclerosis Cognitive Behavioural Screen)	Cognition and behavioural change	Behaviour	Yes	No	No	Yes	2
		Cognition	Yes	No	No	Yes	2
MoCA (Montreal Cognitive Assessment)	Cognition	Cognition	Yes	No	Yes	No	1
Verbal Fluency	Cognition	Cognition	Yes	No	Yes	No	1
CNS-BFS (Centre for Neurologic Bulbar Function Scale)	Physical function	Saliva	No	Yes	No	No	2
CNS-LS (Centre for Neurologic-Lability Scale)	Emotional lability	Behaviour	Yes	No	Yes	No	1
DSM (Diagnostic Statistical Manual) and frontotemporal dementia criteria (FTD)	Diagnosis criteria	Behaviour	No	No	Yes	No	2
Symptom-specific Questionnaires	General	Pain (cramp)	Yes	No	Yes	No	1
		Fatigue	No	No	Yes	No	1
No Data on Assessment Tool	Emergent suicidality	Neuropsychiatric	No	No	No	No	1

^a^Please be aware that this is the number of times used, not the number of trials, as some trials may have utilised several outcome measures to evaluate the same non-motor symptom or a single outcome measure several times

^b^Indicates self-reported cognitive problems, not a formal clinician assessment of cognitive function

**Table 3:** Assessment tool suitability for ALS

Domain	Tool acronym	Tool name	Administrator	Scoring affected by motor disability or speech impairment? (Yes/no)	Disease-specific impairment threshold available? (Yes/no)	Available in languages other than English? (Yes/no)	Time to administer (min)
Quality of life	ALSAQ-5/40	Amyotrophic Lateral Sclerosis Assessment Questionnaire—5 or 40 item	Clinician or researcher	No	Yes	Yes [70]	5–30
Quality of life	SIP/ALS-19	Sickness Impact Profile	Self-report questionnaire	No	Yes	No	10–20
Quality of life	ALSSQOL	Amyotrophic Lateral Sclerosis Specific Quality of Life	Self-report questionnaire	No	Yes	Yes [71]	10–20
Disease burden	ESAS	Edmonton Symptom Assessment Scale	Self-report questionnaire	No	No	Yes [72]	5–10
Quality of life	EQ-5D-5L EQ-5D-3L	EuroQol	Self-report questionnaire	No	No	Yes	5
Quality of life	McGill	McGill	Clinician or researcher	No	Yes	Yes	10–20
Physical	ALS-FRS-(R)	Amyotrophic Lateral Sclerosis Functional Rating Scale	Clinician or researcher	No	Yes	Yes	5–10
Cognition	ACE-III	Addenbrooke’s Cognitive Examination – III	Clinician or researcher	Yes	No	Yes [73]	20–30
Cognition and behaviour	ALS-CBS	Amyotrophic Lateral Sclerosis Cognitive Behavioural Scale	Clinician or researcher and caregiver questionnaire	No	Yes	Yes [74]	5–10
Cognition and behaviour	ECAS	Edinburgh Cognitive ALS Screen	Clinician or researcher and caregiver semi structured interview	No	Yes	Yes [75, 76]	15–30
Behaviour	FBI	Frontal Behavioural Inventory	Caregiver questionnaire	No	No	Yes [77]	5–10
Behaviour	NS	Norris Scale (bulbar sub-scale)	Clinician or researcher	No	No	Yes [78]	10–20
Cognition	VF	Verbal fluency (No data available on type of test)	Clinician or researcher	Yes	Yes	Yes	5–10
Behaviour	CNS-BFS	Centre for Neurologic Studies Behavioural Scale	Self-report questionnaire	No	No	No	10–15
Behaviour	CNS-LS	Centre for Neurologic Studies Lability Scale	Self-report questionnaire	No	No	No	5
Sleep	PSQI	Pittsburgh Sleep Quality Index	Self-report and caregiver questionnaire	No	No	Yes [79]	5–10
Sleep	ESS	Epworth Sleepiness Scale	Self-report questionnaire	No	No	Yes	5
Fatigue	KFS	Krupp Fatigue Severity Scale	Self-report questionnaire	No	No	Yes	5

**Table 4:** Non-motor symptom evaluation summary

Non-motor symptom	Assessment group	Assessment tools used to evaluate	Frequency of use as an outcome measure (% of 237 total trials)
Neuropsychiatric	QoL measures	Sickness Impact Profile, SF-8/12/36, ALS-19, ALSSQOL, ALSAQ-5/40, ESAS, EQ-5D-5L, EQ-ED-3L, McGill	74 (31)
	Symptom-specific	ADI-12, HADS, Hamilton-Depression, Columbia Suicide	
Cognitive	QoL measures	Sickness Impact Profile, ALS-19	16 (6.8)
	Cognitive	ACE-III, ALS-CBS, ECAS, MoCA, DSM Criteria (used as dementia diagnostic criteria), Verbal Fluency	
Behavioural	Quality of life and caregiver burden	Sickness Impact Profile, ALS-19, Zarit Burden Interview, Caregiver Burden Inventory	37 (15)
	Symptom-specific	ALS-CBS, CNS-LS, ECAS, FTD Criteria, Emotionality Analogue Scale, FBI, Norris Scale	
Pain	Quality of life	ALSSQOL, ESAS, EQ-5D-5L/3L, SF-12/36, McGill, ALSAQ-40	55 (23)
	Pain	Cramp Questionnaire, Numeric Rating Scale, Visual Analogue Scale	
Sleep	Quality of life	ALSSQOL, Sickness Impact Profile, Edmonton Symptom Assessment Scale	12 (5)
	Sleep	Visual Analogue Scale, Pittsburgh Sleep Quality Index, Epworth Sleepiness Scale	
Fatigue	Quality of life	ALSSQOL, Edmonton Symptom Assessment Scale	18 7.6)
	Fatigue	Visual Analogue Scale, Presence vs Absence Questions, Krupp Fatigue Severity Scale, Fatigue Severity Scale	
Saliva		ALS-FRS-(R), Visual Analogue Scale, ALSSQOL-R, CNS-BFS	182 (77)

### Symptom evaluation

#### Neuropsychiatric symptoms

Neuropsychiatric symptoms were assessed within quality of life measures in 61 trials (26%); 29 (48%) of these trials used ALS-specific quality of life measures, 29 (48%) generic and 3 a combination. Four trials used a combination of generic quality of life measures and neuropsychiatric assessments that were not ALS specific. No data on assessment tool used were available for one trial.

Seven trials used neuropsychiatric measures which were not developed specifically for people with ALS; ESAS (Edmonton Symptom Assessment Scale [19]), Hamilton-Depression [20], NPI-Q (Neuropsychiatric Inventory Questionnaire [21]) and C-SSRS (Columbia Suicide Severity Rating Scale [22]). Only one trial utilised an ALS-specific neuropsychiatric assessment of depression, the ADI-12 (ALS Depression Inventory [14]), in combination with the more widely used HADS (Hospital Anxiety and Depression Scale [23]), the unmodified version.

Trials evaluating neuropsychiatric symptoms within quality of life measures utilised: Edmonton Symptom Scale (ESS [19]), ALS-Specific Quality of Life (ALSSQOL-R [24]), ALS Assessment Questionnaire (ALSAQ [25]), Short Form Health Survey (SF [26]), EuroQol [27], McGill[28] and Sickness Impact Profile (SIP [29]). These quality of life measures did not provide separate scores for the neuropsychiatric symptoms evaluated. Items focussing on neuropsychiatric symptoms were often limited to binary assessment (present or absent) with scoring reported within the overall quality of life score, making change over time difficult to ascertain (Tables 3, 4).

#### Cognition and behaviour change

Cognition was evaluated in 16 trials (7%); within quality of life measures in 5 trials (Sickness Impact Profile/ALS-19 and ALSSQOL-R). Seven trials used the ECAS (Edinburgh Cognitive Assessment Screen [15]) and one used the ALS-CBS (ALS Cognitive Behavioural Screen [30]), both ALS-specific measures of cognitive impairment. One of the seven trials using the ECAS also evaluated cognition using the MoCA (Montreal Cognitive Assessment [31]), a measure of global cognition that is not specifically designed for people with ALS. Two trials used the ACE-III (Addenbrooke’s Cognitive Assessment [32]) and another a test of verbal fluency, both tests of cognitive function which are not disease specific.

People with ALS may lack insight into cognitive and behavioural changes [33], or downplay experiences due to stigma [34]. Objective measures (such as the ECAS, ALS-CBS, MoCA, ACE-III, and verbal fluency) focus on clinical evaluation and task-based assessments, whereas self-report measures (such as the SIP/ALS-19 and ALSSQOL-R) are reliant on the person with ALS to recognise and disclose their cognitive difficulties.

34 trials (14%) evaluated behavioural symptoms in participants. Five of these were within the context of quality of life measures: SIP and ALS-19. Nine were within assessment tools also evaluating cognition, eight trials using measures such as the ECAS and ALS-CBS which are specifically designed for ALS and one trial using the FBI (Frontal Behavioural Inventory [35]), a non-disease-specific assessment including behavioural items. Emotional lability is a key behavioural change experienced by some individuals with ALS and was evaluated in 19 trials; 18 of which used the Norris scale (which includes one item assessing emotional lability [36]) and one the visual analogue scale on emotionality.

#### Pain

Pain was evaluated in 55 trials (23%). Assessment was included in the context of quality of life measures (such as the ALS Assessment Questionnaire, EuroQoL measures and ALS Quality of Life tools) in 46 trials. Frequency of reporting changes in levels of pain are not reported separately when evaluated in quality of life measures.

Cramp was specifically addressed in trials using questionnaires or visual analogue scales [37]. Other outcome measures evaluating pain utilised numeric rating scales, functional assessments (Edmonton Symptom Assessment Scale [19]) and quantification of pain-related adverse events (NCT03690791).

#### Sleep

13 trials (5.5%) evaluated sleep, 9 of which utilised only quality of life measures. The quality of life measures did not provide a separate score for sleep-related symptoms as the scores were reported as an overall measure of quality of life. The remaining three trials used the symptom-specific Epworth Sleepiness Scale [38], visual analogue scales or the Pittsburgh Sleep Quality Index [39].

#### Fatigue

18 trials (7.6%) evaluated fatigue as an outcome measure. Eight of these trials measured fatigue within quality of life measures and as a result, no separate scores for each non-motor symptom were reported, only the aggregate score for quality of life. One trial utilised the ESAS, a generic tool to document change in patient-reported symptoms. Nine trials evaluated fatigue specifically, utilising visual analogue scales, presence/absence statements, the Krupp Fatigue Severity Scale [40].

#### Problematic saliva

182 trials (77%) reported using the ALS-FRS or ALS-FRS-(R), as an outcome measure that evaluates saliva within the context of physical function. Ten of these trials also utilised additional saliva evaluations: the CNS-BFS (Centre for Neurologic Studies Bulbar Function Scale), a visual analogue scale and the ALSSQOL-R (ALS Quality of Life Revised [41]). No disease and symptom-specific measures of saliva symptoms were included in the trials within this review. Neither the ALS-FRS nor the CNS-BFS provide scores for the severity or frequency of an individuals’ saliva problems.

### Assessment tools

Of the 237 trials included in this study which evaluated non-motor symptoms, 49 versions or combinations assessment tools were used. In this study, we categorised assessment tools as ALS-specific (designed and validated specifically for people with ALS), symptom specific (focussing only on the non-motor symptom under consideration), both (disease and symptoms specific), and generic (evaluating the symptom within a general measure, usually a quality of life questionnaire). Six instruments used were ALS-specific (designed and validated specifically for people with ALS), four were symptom specific, four were both disease and symptom specific, and seven were symptom-generic (evaluating the symptom within a general measure, e.g. QoL, and not specifically evaluating that symptom).

Versions of the ALS-FRS-(R) most frequently utilised (182 trials, 77%). A complete list of the assessment tools used, and the frequency that they are included as outcome measures, is available in Table 2. 208 of the 237 trials (88%) included in this study evaluated one of the listed non-motor symptoms. However, 102 (49%) of these can be accounted for with the use of the ALS-FRS(R) as a primary or secondary outcome to evaluate physical progression, with a single item on hypersalivation.

#### Quality of Life

70 trials (30%) included quality of life assessments as outcome measures. These quality of life measures frequently contained questions on non-motor symptoms such as mood, pain and fatigue. Often these questionnaires include only a single item evaluating the presence of this non-motor symptom, with no additional information on its impact on the individual, severity or change over time [42, 43].

In the trials included in this review, 20% of the times where non-motor symptoms were assessed, this occurred within a quality of life measure, rather than a scale specifically evaluating that symptom. As a result, often no score for the non-motor symptom is reported. Of the 70 trials that used a quality of life assessment as an outcome measure, only 21 included an additional tool to evaluate non-motor symptoms, which was not the ALS-FRS-(R).

A range of quality of life (QoL) measures were used as outcome measures in the trials included in this review. ALS-specific measures; Sickness Impact Profile ALS, ALS Assessment Questionnaires, ALS-Specific Quality of Life enable us to evaluate how the candidate drug affects aspects of the individual’s life most likely to be affected by ALS. Disease- and symptom-specific measures are more likely to be sensitive and specific enough to detect changes, crucial in clinical trials.

However, findings from drug trials using disease-specific measures are limited in their comparability across neurological conditions. In comparison, more general assessments of quality of life and physical functioning such as the Edmonton Symptom Assessment Scale, Short Form Health Surveys, EuroQol measures, Schedule for Individual QoL and McGill enable researchers to compare findings with existing health-related quality of life and disability data, but at the potential cost of evaluating disease-specific impairment.

#### Symptom-specific measures

Neuropsychiatric outcome measure such as the NPI-Q are neither disease-specific nor symptom specific. Whilst useful to capture the potential presence of broad range of disorders, the utility of this measure to detect change over time is limited due to the dichotomous outcome of Yes/No to the presence of disorders. Symptom-specific measures such as the HADS, C-SSRS and HAM-D were used as outcome measures in other included trials, the suitability of these measures for people with ALS is uncertain, due to overlap with somatic symptoms and disease progression. This can be mediated through the use of revised disease-specific thresholds of impairment [16]. The ADI-12 is a brief measure of depressive symptoms, specifically designed and validated for people with ALS [14]; evidence base and comparability outside ALS is limited.

The ECAS and ALS-CBS are disease-specific measures of cognitive and behavioural symptoms in people with ALS. Designed and validated for use in this population, they focus on the aspects of cognition and behaviour which are most affected in this condition and are sensitive to detecting changes across repeated assessment [44]. The MoCA and ACE-III are measures of global cognition, whilst not specifically intended to evaluate cognitive impairment in people with ALS they may have utility as outcome measures in trials to detect potential changes. However, both of these assessments rely on drawing tasks to evaluate cognitive functioning, the scoring on which may be detrimentally affected by the physical progression characteristic of ALS.

Verbal fluency is a measure of a specific aspect of cognition, often affected in people with ALS, however, using this assessment in isolation may be insufficient to detect the broad range of cognitive function that can be affected by ALS progression.

As ALS exists on a disease spectrum with frontotemporal dementia (FTD) [45], measures of behaviours that are included in FTD diagnosis, such as the Frontal Behavioural Inventory and DSM criteria (Diagnostic and Statistical Manual), can be of relevance to evaluating behavioural symptoms in people with ALS, even those who do not meet diagnostic threshold for FTD. However, these measures may also miss the nuanced behaviours that can occur in the heterogeneous presentations of ALS. Emotional lability can be a commonly experienced symptom of bulbar dysfunction, measures such as the CNS-LS, are beneficial to evaluate disease- and symptom-specific outcomes.

Pain was primarily evaluated within quality of life measures, both ALS-specific and generic measures. Symptom-specific assessments of pain and cramp prevalence and severity were limited to visual analogue scales and Cramp Questionnaires, which may not be sufficiently objective to detect the nuanced changes occurring within the progression of ALS and the potential impact of a candidate drug.

Symptom-specific sleep measures used in the trials in this review are the Epworth Sleepiness Scale and Pittsburgh Sleep Quality Index are beneficial to understand participant perspective and acknowledge that sleep quality is greater than just time spent at rest. However, in ALS, disordered breathing and declining respiratory function can be a significant contributor to the multifactorial issue of sleep. In tools which are not ALS specific, the impact of respiratory symptoms may not be accounted for. In ALS, the Epworth Sleepiness Scale has the additional benefit of indicating the severity of respiratory symptoms.

Contribution of other symptoms and evaluation using symptom-specific scales is also of consideration when evaluating fatigue. Using symptom-specific outcome measures such as the Krupp Fatigue Severity Scale, and the FSS, may not reflect the interwoven contributions to the conceptualisation and causes of physical and mental fatigue experienced in ALS.

#### Saliva assessments

Whilst 184 (78%) trials evaluated saliva, in these trials saliva was assessed in the context of a single sub-domain score of the ALS-FRS(R) or with other bulbar symptoms, alongside swallowing and speech, in the CNS-BFS. Impact of the candidate drug, separate saliva score and change in saliva problems were not reported.

ALS-FRS-(R) includes a single item on hypersalivation. CNS-BFS also considers problematic saliva, and the Norris Scale an item on behavioural change. As a result, despite apparent frequent measurement of saliva, as the measurement is within the ALS-FRS-(R), we know little about the potential impact of these candidate drugs on the saliva symptoms which can have a significant affect upon people with ALS [6].

Of the 206 trials which included an assessment of any non-motor symptom, saliva (using larger physical function, quality of life or bulbar assessment tools) was the only non-motor symptom assessed in 102 (49%) of these trials. Ultimately, excluding the use of the ALS-FRS(R) as a physical function outcome measure, including the single item on salivation, saliva problems were under-evaluated. Only 80 (44%) of the 182 trials using the ALS-FRS-(R), evaluating saliva, assessed any additional non-motor symptom.

## Discussion

### Overview

As our conceptualisation of ALS broadens from a motor-only disorder to one of multi-system involvement, it is vital that clinical management guidelines and trial design continue to reflect this. Effective symptom management remains a major priority for ALS care, as stated in NICE 2016 care guidelines [8]. Clinical management and trial design guidance recommend the inclusion of symptom-focussed outcome measures to evaluate potential additional therapeutic benefits [9, 10].

This study considers the evaluation of non-motor symptoms as outcome measures in 237 trials proposed to include over 29,222 trial participants with ALS in the last 25 years, since the licensing of riluzole in 1994. The non-motor symptoms included in this review are neuropsychiatric symptoms, cognitive and behavioural changes, pain, disordered sleep, fatigue, and problematic saliva, all of which are prevalent in and impactful in ALS [1]. Our findings indicate that non-motor symptoms were not consistently evaluated and where evaluated, assessment tools were not specific to ALS, or the non-motor symptom being evaluated.

### Evaluation and management

Effective management and treatment of non-motor symptoms can have a significant impact on the lives of people with ALS and their caregivers, reducing disease burden and improving quality of life [8]. Non-motor symptoms can benefit from both non-pharmacological and pharmacological interventions and in conditions such as ALS where symptom management is currently the primary focus, managing these non-motor symptoms can benefit those living with ALS [46]. Using non-motor assessments in clinical evaluation of people with ALS can help us address these symptoms in care planning, disease management and when designing future research.

Under-evaluation of non-motor symptoms using disease specific measures is a potentially missed opportunity when considering the holistic impact of drug candidates on these troublesome symptoms. In our previous work, we found that neuropsychiatric and cognitive symptoms were consistently under-assessed in ALS trials [11]. Whilst improvement in motor functioning and prolonging survival remain the main goal in clinical drug trials, additional symptomatic benefit of candidate drugs can be of great interest. An additional benefit of including measures to evaluate these non-motor symptoms in trial design is a better understanding of the potential negative impact of candidate drugs on these aspects of ALS. An investigative medicinal product that may result in, or worsen existing, non-motor symptoms and in turn increase disease burden, may offset the potential improvement in motor symptoms for people with ALS. Greater knowledge of these side effects can help to inform licensing decisions and future suitability for prescription of the medication to sub-groups of the ALS population.

### Assessment tools

In other neurodegenerative conditions, such as Parkinson’s disease, where non-motor symptoms are common and impactful, disease-specific and symptom-specific scales such as Parkinson’s Disease Fatigue Scale [47], King’s Parkinson’s Disease Pain Scale [48] and Parkinson’s Disease Sleep Scale [49] have clinical utility [50]. As a result, in this review we also considered the intended purpose of each of the tools utilised to evaluate non-motor symptoms. In using assessment tools that are specific to, or adapted for, the population we are evaluating, we are better able to determine the prevalence and progression of non-motor symptoms, whilst accounting for the progressive disability and speech impairments of ALS that may influence responses. Including revised impairment thresholds in well-established generic measures can also be a viable alternative to mitigate potential confounding effects of ALS.

However, measures designed to evaluate the non-motor features of ALS and other neurodegenerative diseases have not been frequently employed in clinical care, research or trial design [10]. Potential barriers to their use include a limited evidence base compared to established measures and additional time burden for participants. These obstacles can be addressed with further research into the validity, reliability and utility of brief measures adapted to assess these non-motor symptoms within the context of neurodegenerative diseases.

### Strengths and weaknesses of the study, and future recommendations

This study indicates that non-motor symptoms have not been comprehensively or consistently evaluated within clinical trials of ALS. An improved understanding of the frequency that these symptoms occur, and their contribution to acquired disability, will enable us to provide a more holistic overview of an ALS diagnosis and potential impact of investigative medicinal products. The key strength of this study is that it provides a comprehensive evaluation of ALS trials completed, published or registered since 1994. In addition, we provide a detailed overview and critique of the assessment tools used to explore these non-motor symptoms in the included trials. However, a weakness of the current work is the focus on a limited number of non-motor symptoms in ALS, namely neuropsychiatric, cognitive and behavioural changes, pain, disordered sleep and fatigue and problematic saliva. However, the scope of non-motor symptoms can be extended in future studies to assessment of other symptoms including gastrointestinal issues, dysphagia and sexual dysfunction.

We recommend that future clinical trials should include non-motor outcome measures. In addition, more research should focus on the association between these symptomatic outcomes and the potential benefit experienced by trial participants. Whilst it is appropriate that measurement of change in functional decline and improvement in survival remain as primary outcomes in confirmatory trials, we recommend future trials include disease-specific secondary outcome measures to establish the effect of investigative medicinal products on non-motor symptoms to enable a more complete profile of how a candidate drug may affect pwALS. Cognitive assessments are already gradually receiving greater prominence in trial design [11], reflective of Airlie House guidance encouraging the use of cognitive or behavioural functioning as primary or secondary outcome measures [9].

Evaluation of the key non-motor symptoms considered in this study was primarily using tools which may not be suitable for people with ALS, or generic assessments of physical function or quality of life where symptom-specific changes were not apparent. These non-motor symptoms should be evaluated with assessment tools which are ALS-specific or validated for use in people with ALS, including disease-specific impairment thresholds where possible.

Future work should focus on evaluating the prevalence and impact of each of these non-motor symptoms in people with ALS. This research should also explore the comparison of different assessment tools for each non-motor symptom. Providing recommendations for assessment tools that are suitable to evaluate non-motor symptoms, or the availability of disease-specific impairment thresholds, will be a useful and relevant direction for future work.

## Supplementary Information

Below is the link to the electronic supplementary material.

Supplementary file1 (XLSX 76 kb)

## Data Availability

Supplementary Material Document 1.
